# Detecting Climate Driven Changes in Chlorophyll-a Using High Frequency Monitoring: The Impact of the 2019 European Heatwave in Three Contrasting Aquatic Systems

**DOI:** 10.3390/s21186242

**Published:** 2021-09-17

**Authors:** Gary Free, Mariano Bresciani, Monica Pinardi, Claudia Giardino, Krista Alikas, Kersti Kangro, Eva-Ingrid Rõõm, Diana Vaičiūtė, Martynas Bučas, Edvinas Tiškus, Annelies Hommersom, Marnix Laanen, Steef Peters

**Affiliations:** 1 Institute of Electromagnetic Sensing of the Environment, National Research Council of Italy (CNR-IREA), via Bassini 15, 20133 Milan, Italy; bresciani.m@irea.cnr.it (M.B.); pinardi.m@irea.cnr.it (M.P.); giardino.c@irea.cnr.it (C.G.); 2 Tartu Observatory, University of Tartu, Observatooriumi 1, Tõravere, 61602 Tartu, Estonia; krista.alikas@ut.ee (K.A.); kersti.kangro@ut.ee (K.K.); 3 Chair of Hydrobiology and Fishery, Institute of Agricultural and Environmental Sciences, Estonian University of Life Sciences, Kreutzwaldi 5, 51006 Tartu, Estonia; room@emu.ee; 4 Marine Research Institute, Klaipėda University, Universiteto Ave. 17, 92294 Klaipėda, Lithuania; diana.vaiciute@jmtc.ku.lt (D.V.); martynas.bucas@jmtc.ku.lt (M.B.); edvinas.tiskus@apc.ku.lt (E.T.); 5 Water Insight, Fahrenheitstraat 42, 6716 BR Ede, The Netherlands; hommersom@waterinsight.nl (A.H.); laanen@waterinsight.nl (M.L.); peters@waterinsight.nl (S.P.)

**Keywords:** lake, lagoon, climate change, high-frequency monitoring, WISPstation, chlorophyll-a, Cyanobacteria blooms, phytoplankton

## Abstract

The frequency of heatwave events in Europe is increasing as a result of climate change. This can have implications for the water quality and ecological functioning of aquatic systems. We deployed three spectroradiometer WISPstations at three sites in Europe (Italy, Estonia, and Lithuania/Russia) to measure chlorophyll-a at high frequency. A heatwave in July 2019 occurred with record daily maximum temperatures over 40 °C in parts of Europe. The effects of the resulting storm that ended the heatwave were more discernable than the heatwave itself. Following the storm, chlorophyll-a concentrations increased markedly in two of the lakes and remained high for the duration of the summer while at one site concentrations increased linearly. Heatwaves and subsequent storms appeared to play an important role in structuring the phenology of the primary producers, with wider implications for lake functioning. Chlorophyll-a peaked in early September, after which a wind event dissipated concentrations until calmer conditions returned. Synoptic coordinated high frequency monitoring needs to be advanced in Europe as part of water management policy and to improve knowledge on the implications of climate change. Lakes, as dynamic ecosystems with fast moving species-succession, provide a prism to observe the scale of future change.

## 1. Introduction

Lakes provided the field of science with the initial inspiration for the concept of an ecosystem and globally represent a vital resource in terms of water supply and other ecosystem services [1,2]. Water resources are currently threatened by the negative impacts deriving from anthropogenic development and alteration, population growth, and climate change [3,4]. A main challenge for researchers is to deepen the knowledge on these impacts and their implications for aquatic environments [5]. At a global scale, the increasing eutrophication and water pollution in rivers, lakes, coastal, and marine environments is feeding the request for environmental monitoring based on innovative and advanced methods and systems especially in water quality monitoring [6]. As an example, nutrient enrichment triggers a progression of eutrophic responses, such as the increase of phytoplankton biomass which is related to and rapidly responds (i.e., hourly/daily) to nutrient loads into inland and coastal waters [7,8]. It is well established that, in general, nutrient concentration [9], light condition, water temperature [10], and predation [11] are the main factors that affect the phytoplankton proliferation in aquatic ecosystems. Zhang et al. [12] summarized that phytoplankton biomass variation and its long-term trends are influenced by several processes, such as nutrients and their ratio, photothermal processes (light availability and temperature), and water mixing events, together with climate-related variables (wind speed and underwater available light) that play also an important role as predictors. Temperature and irradiance interaction and its effects on phytoplankton growth have been studied in many experiments (e.g., [13,14]), but their relationships are still under debate [15]. The identification of intraday variability of phytoplankton abundance is critical in tidal habitats and in shallow lakes due to the fact that long-term trends and time-averaged quantities can be driven by interactions occurring at higher frequencies [16,17,18].

In the European Union, the Water Framework Directive (WFD) aims to stop deterioration and restore surface water to at least good status. The achievement of this objective is through a river basin management plan detailing the program of measures to be carried out, the success of which is determined by monitoring [19,20]. Monitoring, therefore, forms a pivotal part of the process and many practitioners have identified improvements in the temporal and spatial resolution as a development need of the directive [21].

One of the main advantages of high frequency in situ sampling is the chance of having different quality elements measured with reliable and accurate methodologies in the field and in the laboratory [22,23]. On the other hand, in situ monitoring through conventional procedures (e.g., by boat as routinely carried out to fulfill the requirements of the WFD) has a limited spatial and temporal coverage, and lacks the availability and timeliness of near real-time data acquisition [24]. Such infrequent information will probably fail to accurately represent the dynamic nature of many aquatic ecosystems.

At present, different sensors mounted on different platforms (e.g., fluorimeters mounted on buoys) or on probes (e.g., CTD -conductivity, temperature, and depth) are used to provide continuous real-time information on water quality parameters (e.g., [24,25,26]). In addition, in recent years, different studies showed the possibility to use in situ radiometric sensors to characterize water quality status and its temporal variability by means of continuous measurements (e.g., [27,28]). The main parameters retrieved by hyperspectral sensors are chlorophyll-a (Chl-a) and secondary pigments concentration, a proxy of phytoplankton biomass, suspended solids, and CDOM (colored dissolved organic matter). In situ high-frequency monitoring (HFM) and subsequent data analysis allow for the selection of the optimal sampling time on the basis of the seasonal evolution of different water bodies [29].

A step forward is the integration of in situ spectroradiometer and satellite data to provide spatial and temporal information for water monitoring and management [27,30]. In this way, satellite remote sensing data can be used to retrieve Chl-a maps for water quality monitoring going beyond the limited spatial coverage of in situ sampling [31]. This will also help in the extrapolation of seasonal and annual dynamics and short temporal variations which involve hourly and daily cycles, enabled by continuous data recording from radiometers [32,33].

The influence of climate change on lakes is becoming increasingly concerning worldwide. The increase in summer temperatures has been estimated at 0.34 °C per decade with lake specific parameters like morphology contributing to the diversity of response at the regional level [34]. Often, the influence of climate change on the structural functioning of the lake or catchment is more important than its direct physiological effects [35]. While phytoplankton can show a direct response in growth in relation to temperature [36,37], structural change has been observed in deep lakes where increased winter temperatures have reduced the mixing of lake layers. This has resulted in reduced nutrients leading to smaller spring blooms, thereby altering the seasonal phenology of the phytoplankton [38,39]. Meanwhile, at higher latitudes longer growing seasons have been attributed to a higher proportion of rain compared to snow resulting in an earlier delivery of the nutrients to lakes [40].

Of particular concern is the predicted increase in potentially harmful summer blooms of cyanobacteria with climate change and eutrophication [15,16,17]. In Europe, several heatwave events have been associated with increased blooms. In 2003, a summer heatwave bloom event in the Nieuwe Meer lake was the result of an increased direct growth of cyanobacteria as well as the more stable water column under which buoyant cyanobacteria have a competitive advantage [37]. In contrast, comparing 2003 with 2006 in lake Müggelsee, which had relatively equal hot summers, only 2006 saw a significant bloom, which was attributed to more stable stratification in 2006 supported by calmer wind. When comparing the influence of heatwaves on multiple lakes the different nutrient statuses, mean depth, and residence time are likely to be of key importance [41,42].

It is now virtually certain that warming will continue with an over 90% likelihood that there will continue to be an increase in the frequency of heat extremes over the 21st century in Europe, especially in southern regions [43]. Recent projections have predicted that lakes will get warmer for longer periods, with heatwaves possibly extending across multiple seasons [44]. In July 2019, a heatwave occurred in Europe with record daily maximum temperatures over 40 °C observed in several places. Temperatures were locally 6 to 8 °C higher than the average warmest day of the year for the period 1981–2010 [45]. Here, we examine chlorophyll-a data from two lakes and one lagoon in Europe, monitored at high frequency using a WISPstation, to examine daily variation and focus on the period comprising the summer heatwave in July 2019. We expect to see a response of phytoplankton, as indicated by chlorophyll-a, across the three contrasting sites to this heatwave event.

## 2. Study Areas

**Figure 1:**
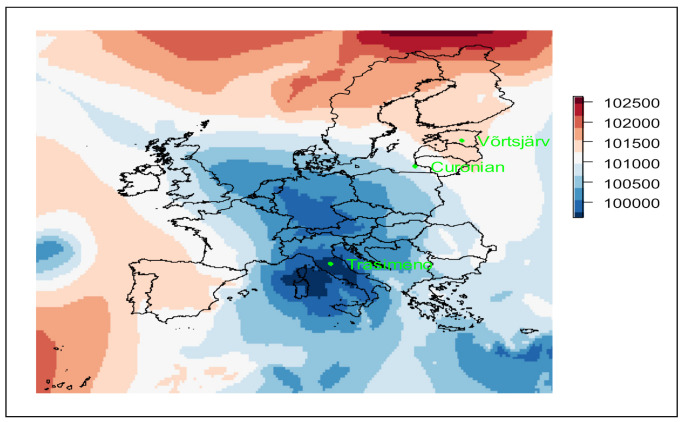
Map of Europe showing the three WISPstation sites together with atmospheric pressure (Pa) on the 28 July 2019 indicating the weather system that marked the end of the July heatwave.

Three shallow inland waters with recurrent algal blooms located in different parts of Europe were considered. Two of the three areas share a humid continental climate (Curonian Lagoon in Lithuania, and Lake Võrtsjärv in Estonia) and the third one, Lake Trasimeno (Italy), is characterized by a Mediterranean climate according to the Köppen-Geiger climate classification [46]. In Figure 1 and Table 1, the locations and main features of the three study areas, respectively, are reported.

### 2.1. Lake Trasimeno

Lake Trasimeno is a shallow meso-eutrophic lake with a fluvial and post-tectonic origin, located in central Italy (43.13° N; 12.10° E). It is the fourth largest lake in Italy (surface area 120.5 km^2^) and has a circular shape, with a maximum depth of 6.3 m [47]. Lake Trasimeno is generally turbid (average Secchi disk depth 0.9 m) with an annual average total phosphorus concentration of 27 µg L^−1^ for the years 2015–2017, while the annual average of chlorophyll-a (Chl-a) ranged from 5.4 to 14.7 mg m^−3^ for this period [48]. The water column is unstratified, with recurrent sediment resuspension due to wind action. According to the WFD system of classification, the lake is currently classified at moderate ecological status [19,20,48,49]. Lake Trasimeno has a phytoplankton assemblage dominated by chlorophytes and dinoflagellates. Cryptophytes can also comprise a relatively large portion of the biomass, whereas euglenophytes and diatoms are relatively scarce [50]. In summer, the high nutrient availability favors the occurrence of phytoplankton blooms, including cyanobacteria species (e.g., *Cylindrospermopsis raciborskii*, *Planktothrix agardhii*) [50,51].

### 2.2. Curonian Lagoon

The Curonian Lagoon (55.22° N, 21.06° E) is a large, shallow water body (total area 1584 km^2^, mean depth 3.8 m). Geographically, the lagoon is positioned between the Republic of Lithuania and the Russian Federation. The lagoon is located at the interface between the south-eastern Baltic Sea and the watershed of the Nemunas River. The seasonally variable mixing of fresh and brackish water masses creates spatially and temporally unstable gradients with a salinity that ranges from 0 to 7 PSU. The Nemunas River runoff (nearly 23 km^3^ year^−1^) contributes approximately 96% of the total riverine runoff, and 77% of the water balance of the lagoon. Water renewal time is typically 10–40 days during periods of elevated river discharge, and increases to 60–100 days during low discharge [52,53]. At present, the Curonian Lagoon is considered to be eutrophic or hyper-eutrophic and, according to the WFD system of classification, the lagoon has been classified at moderate to bad status. The main ecological problem is eutrophication due to pollution by nutrients with diatom blooms in spring and wide-spread cyanobacteria blooms in summer with recurring cyanobacteria surface accumulations (scums) mainly caused by *Aphanizomenon flosaquae*, *Microcystis* spp., *Dolichospermum* spp. and *Planktothrix agardhii*. The concentration of Chl-a ranges from 4 to more than 400 mg m^–3^ [54,55,56] and total phosphorus ranges from around 34 µg L^−1^ in spring to around 211 µg L^−1^ in summer [57].

### 2.3. Lake Võrtsjärv

Lake Võrtsjärv (58.28° N, 26.03° E) is a turbid, well-mixed, eutrophic, and non-stratified lake with high CDOM, phytoplankton and total suspended matter (TSM). The lake’s ecosystem is strongly physically controlled due to its large surface area (270 km^2^) and shallow depth (mean depth 2.8 m) with the average amplitude of water level fluctuations reaching 1.34 m [58]. According to the WFD system of classification, the lake has been classified predominantly as having a moderate status. The annual mean total phosphorus concentration is around 40 µg L^−1^ and Chl-a about 35.7 mg L^−1^ [59]. Cyanobacteria and diatoms dominate the algal groups, whereas green algae, cryptophytes, and dinoflagellates are scarce. Cyanobacteria *Limnothrix planctonica* and *L. redekei* dominate during the entire year [60].

## 3. Materials and Methods

**Figure 2:**
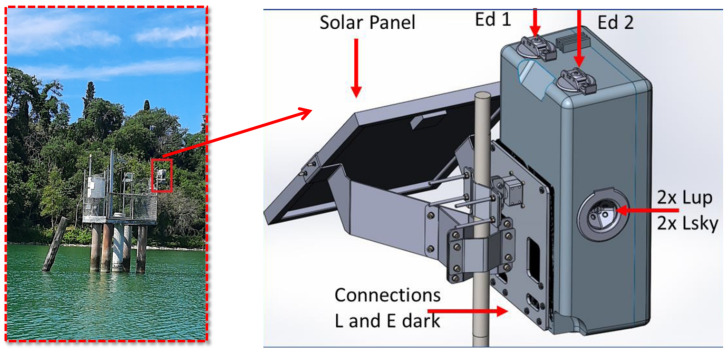
Photograph of the WISPstation in Lake Trasimeno (Italy). Red box in photo indicates the layout and installation of the instrument which is further detailed in the diagram on the right from Peters et al. [61] as follows: 2 Radiance channels collecting Lup and Lsky in the NNW direction; 2 Radiance channels collecting Lup and Lsky in the NNE direction; 2 Irradiance channels (Ed); 1 unexposed dark radiance (L) channel for evaluation of radiance channel degradation; 1 unexposed dark irradiance (E) channel for evaluation of the degradation of irradiance channels.

The high-frequency Chl-a concentration data were gathered from a fixed position autonomous optical measurement device, the WISPstation deployed in Lake Trasimeno (WISPstation001; 43.1223° N, 12.1344° E), Lake Võrtsjärv (WISPstation005; 55.4127° N, 21.10027° E) and halfway down the Curonian lagoon (WISPstation006; 58.2112° N, 26.1080° E) (cf. Figure 1). The WISPstation measures the radiance (5 channels) and irradiance (3 channels) in the spectral range of 350–900 nm with a spectral resolution of 4.6 nm. The WISPstation design is based on a fixed viewing angles system: the azimuth angle is optimal around 138 degrees from the sun for radiance measures, Lup and Lsky angles are 42 degrees from the nadir and from the zenith, respectively, to avoid sun glint (Figure 2). In the Northern hemisphere, the instrument should be installed looking in a northward direction with two sets of sensors looking NNW and NNE to provide two optimal viewing geometry moments during the day and a large time window with acceptable ones. This configuration allows the hyperspectral remote sensing reflectance (Rrs) to be derived. Consequently, some of the most important bio-physical water quality parameters, such as chlorophyll-a, cyanobacteria pigment phycocyanin, suspended matter, presence of scums, and transparency can also be derived [51]. A regular measurement cycle takes less than one minute depending on ambient light conditions, so the measurement frequency can be set in orders of minutes. Recorded data are transmitted to the database (“WISPcloud”) autonomously through a 3G connection. Moreover, the instrument can be remotely accessed for update and configuration of measurement frequency. It is autonomously powered by a solar panel and internal large battery. More details can be found in Peters et al. [61] and Bresciani et al. [28].

The Rrs data from the three WISPstations between July and December 2019, collected every 15 min, allowed the Chl-a concentrations to be derived according to a standard water quality algorithm appropriate for turbid eutrophic water [62], which makes use of a reflectance band ratio at 704 and 672 nm with backscattering derived from the reflectance at 776 nm. A validation exercise carried out across the three countries as part of the project had an R^2^ of 0.90 and a slope of 0.91 between 29 sets of Chl-a estimates derived from the WISPstation and simultaneous Chl-a determined from laboratory measurements (Supplementary Materials Figure S1) (further details in [63]).

Meteorological data on wind speed and direction, precipitation, air temperature, and solar irradiation were obtained from local meteorological stations. Seven-day antecedent rain total, and seven-day average wind speed and temperature were also calculated for analysis.

**Figure 3:**
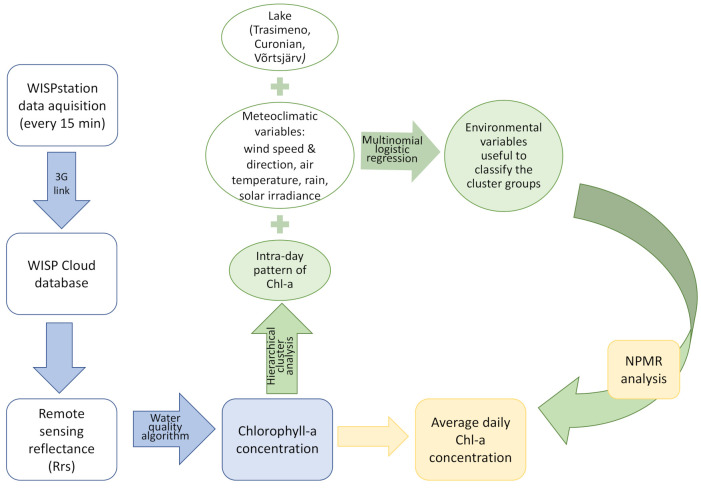
Schematic diagram of data acquisition and analysis carried out.

In order to identify and aggregate similar patterns in the diurnal behavior of Chl-a, a hierarchical cluster analysis was carried out using Sørensen distance with flexible beta linkage [64]. Analysis was carried out on daily data between 9:00 and 16:00 relativized to the maximum concentration. This was done so that the daily pattern, rather that the differences in absolute concentrations, was focused on. Cluster end groups were assigned a number based on the row number of the first item assigned to that group. Multinomial logistic regression was used to see if environmental variables could correctly classify the different cluster end groups using the nnet package in R [65]. This was used in preference to discriminant analysis so that the categorical variable “lake” could be included in the analysis. After the removal of autocorrelated variables (r ≥ 0.6), a total of five remained for analysis: solar irradiance, air temperature, wind speed, precipitation, and lake. Wind speed rather than wind components was chosen for analysis as it allows comparison across the two different lakes whereas wind components may reflect local conditions affecting wind direction. As n was small for some clusters, the data was not partitioned into training and test sets. A schematic diagram of the data acquisition and data analysis carried out is shown in Figure 3.

Nonparametric Multiplicative Regression (NPMR) [66] was used to estimate the response of average daily Chl-a concentration to climate and the environmental parameters listed above. NPMR can define response surfaces using predictors in a multiplicative rather than in an additive way. This method is progressive in better defining unimodal responses than other methods such as multiple regression [66]. It has previously been applied to model tree species distribution [67], the response of lichens to climate change [68], and in time-series analysis [69]. NPMR was applied using the software HyperNiche version 2.3 [70]. The response of Chl-a was estimated using a local mean multiplicative smoothing function with Gaussian weighting. NPMR models were produced by adding predictors stepwise with fit expressed as a cross-validated R^2^ (xR^2^) which can be interpreted in a similar way as a measure of fit like a traditional R^2^. The sensitivity, a measure of influence of each parameter included in the NPMR model, was estimated by altering the range of predictors by ±0.05 (i.e., 5%) with resulting deviations scaled as a proportion of the observed range of the response variable. Sensitivity can be used to evaluate the relative importance of variables included in models because NPMR models are unlike linear regression and have no fixed coefficients or slopes. Instead, model specification is achieved by listing six items: (1) the data used, (2) statement of the local model used (i.e., local mean), (3) statement of the weighting function (Gaussian weighting), (4) the independent variables used, as listed above, (5) variable type used—here quantitative rather than categorical and (6) the tolerance values used, reported in Section 4. Further details including the detailed mathematical formula along with worked examples have been published [66,69,71]. These options selected together with the data used allow repetition of the analysis.

## 4. Results

The structure of the analysis carried out is to first examine the water spectral signatures, then the daily variation in estimated Chl-a and subsequently, that of the summer period across the three sites to explore and contrast the response and reasons for the differences.

**Figure 4:**
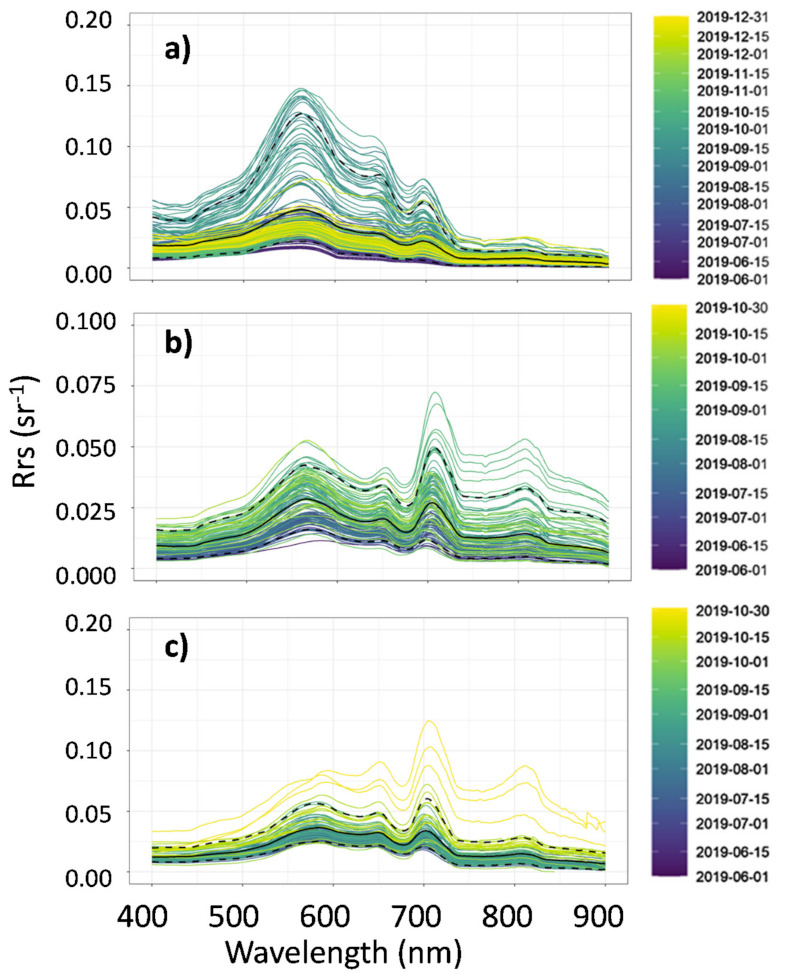
Daily averages of Remote sensing reflectances (Rrs) for the period of measurement in Lake Trasimeno from 1 June to 31 December 2019 (**a**), Curonian Lagoon (**b**), and Lake Võrtsjärv (**c**) from 1 June to 31 October 2019. Black continuous line is the Rrs mean value computed over the entire period and black dotted lines are the relative 5th and 95th percentiles.

The daily values of Rrs, recorded by the three WISPstations during the period of measurement, are shown in Figure 4. Specifically, Lake Trasimeno (Figure 4a) had the highest Rrs values in terms of magnitude in the green and red regions in the autumn period, which are characteristic of the lake’s high turbid waters due to continuous wind-induced bottom sediment resuspension processes. The spectral signature of the Curonian Lagoon (Figure 4b) displayed the typical shape of reflectance in the spectral region between 625 and 650 due to the presence of phycocyanin. In addition, during the days characterized by a high cyanobacteria bloom or scum, the Rrs values were very high in the near-infrared (NIR) region. In Lake Võrtsjärv (Figure 4c), the spectral signatures recorded indicate the high trophic level of the lake, similar to the other aquatic ecosystems investigated. Specifically, there is an evident effect of high absorption due to phytoplankton Chl-a between 670 and 680 nm with the consequent reflectance peak in the region from 700 to 710 nm.

**Figure 5:**
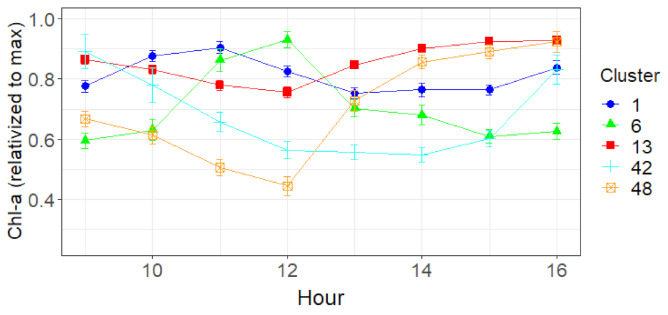
Average hourly (UTC time) Chl-a (with standard error), relativized to maximum value per day.

**Figure 6:**
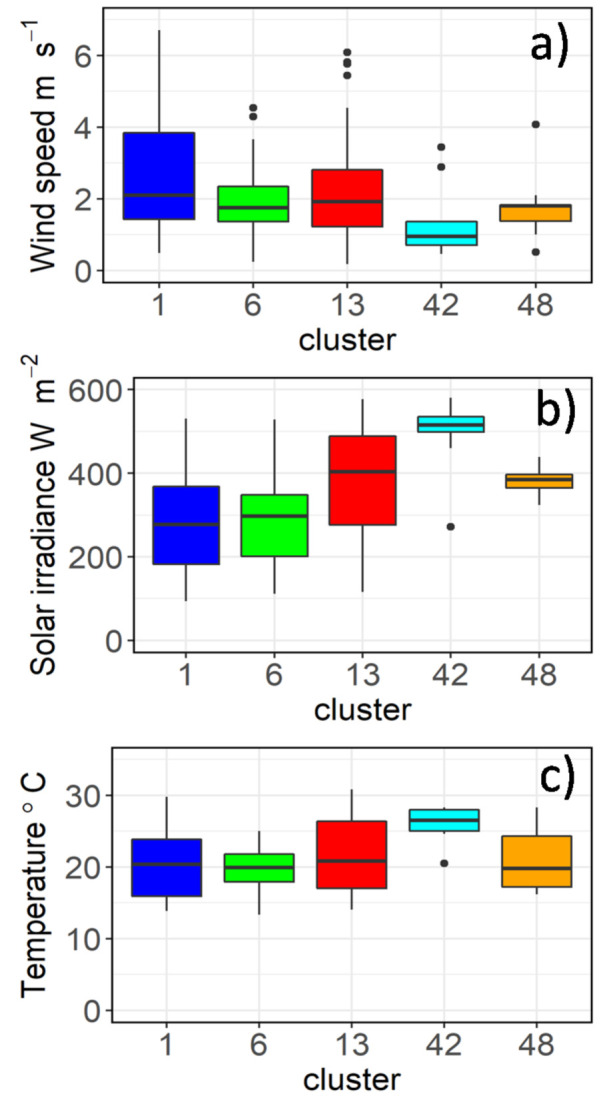
Boxplots of environmental parameters (**a**) wind speed, (**b**) solar irradiance and (**c**) air temperature for the different clusters.

To explain the dynamic changes of Chl-a occurring within a day we examined the hourly variation. It was apparent that there was no consistent daily pattern. A cluster analysis of the hourly Chl-a data by day (June–August 2019) was carried out to group the diurnal patterns. Data was included only where it was available for the three lakes on the same day in order to ensure a balanced comparison. Five clusters were identified (Supplementary Materials Figure S2) and their average pattern was graphed (Figure 5). Cluster 48, 42, and 13 all had a u or v shape with a decline until 12:00 followed by an increase. However, for clusters 1 and 6, the pattern is one of an increase until 11:00 or 12:00 followed by a decline. In addition, clusters 1 and 13 were more stable and showed less change compared to the other clusters. The three clusters (48, 42, 13) that had a u or v shaped daily Chl-a pattern had a tendency to have higher solar irradiation compared to clusters 1 and 6 (Figure 6). Two of these clusters (48, 42) also tended to have a lower wind speed, while there were no discernible differences in air temperature between the clusters (Figure 6).

Multinominal logistic regression was carried out to better identify the environmental variables that may play a role in determining the different daily patterns. Coefficients for environmental parameters that were significant (*p* ≤ 0.05), as tested by two-tailed Wald z tests, for the different clusters included solar irradiance (cluster 42), wind speed (cluster 42), and lake (clusters 13,48). However, we had limited success using multinomial logistic regression to reclassify clusters using the environmental variables, with correct classifications ranging from 0% for clusters 48 and 6 to 66% for cluster 13 (Table 2).

**Figure 7:**
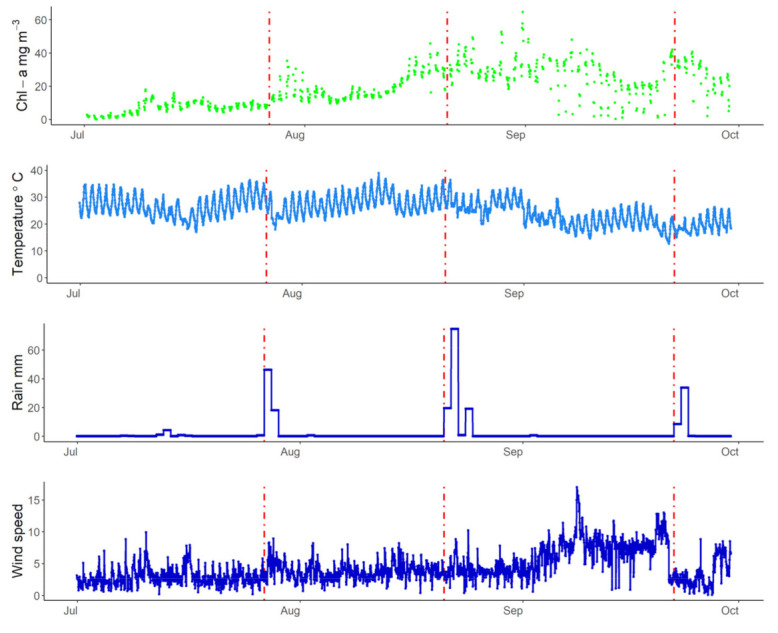
Lake Trasimeno Chl-a (hourly), air temperature (hourly), rain (daily total), and wind speed (hourly) between July and September 2019. Dashed red lines indicate 27 July, 21 August, and 22 September 2019.

**Figure 8:**
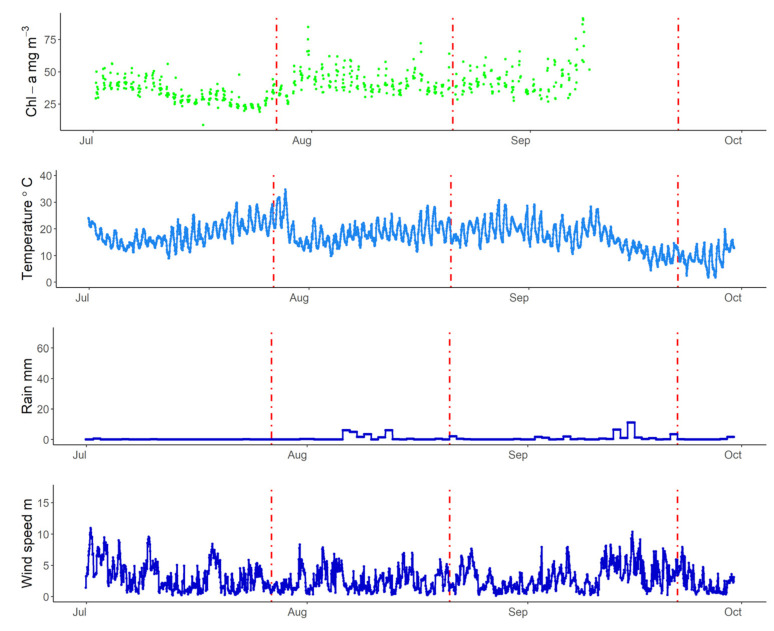
Lake Võrtsjärv Chl-a (hourly), air temperature (hourly), rain (daily total), and wind speed (hourly) between July and September 2019. Dashed red lines indicate 27 July, 21 August, and 22 September 2019.

**Figure 9:**
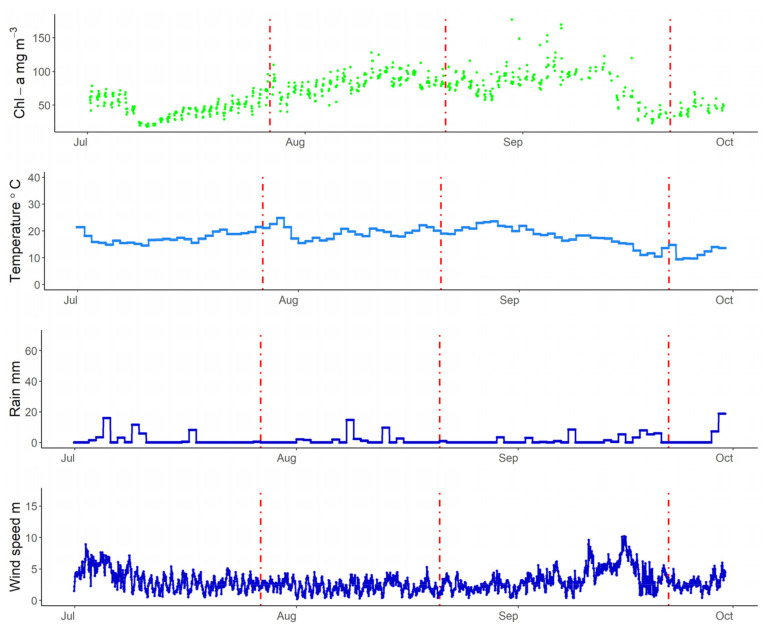
Curonian Lagoon Chl-a (hourly), air temperature (hourly), rain (daily total), and wind speed (hourly) between July and September 2019. Dashed red lines indicate 27 July, 21 August, and 22 September 2019.

July 2019 was one of the warmest Julys on record in Europe [72]. The increase in air temperature during July for the lakes is visible in Figure 7, Figure 8 and Figure 9. In Lake Trasimeno the peak temperature for July occurred on the 24 July reaching 36.4 °C. This warm period then ended when an area of low pressure moved northwards (see air pressure map in Figure 1). This resulted in a sudden drop in air temperature from 32 °C on 27 July (indicated by a red line in Figure 7) down to 18.0 °C on 28 July. This coincided with increased rainfall and wind speed. This was followed by a notable increase in Chl-a in Trasimeno. Similarly for Võrtsjärv (Figure 8), with a one day delay, air temperatures dropped from an annual peak of 34.8 °C on 28 July to 15 °C on 29 July. This was accompanied by increased wind speed, but only minor rainfall compared to Lake Trasimeno, followed by an increase in Chl-a. For the Curonian Lagoon (Figure 9), the air temperature was only available at daily resolution and therefore less comparable but declined from 24.9 °C on 29 of July to 15.5 °C on 1 August. There was only minor variation in rainfall and wind speed associated with this event and Chl-a linearly increased throughout August thereafter. In contrast to this linear increase in the Curonian Lagoon, in Trasimeno and Võrtsjärv lakes this event marked a more distinct shift to higher Chl-a concentrations which remained elevated for the following months compared to July.

Apart from this late July event, there were several other concordant patterns between weather and WISPstation estimated Chl-a in Lake Trasimeno. A rainfall event on 21 August was accompanied by an increase in Chl-a as was a subsequent rainfall event on 22 September, however, this event was also accompanied by a notable drop in wind speed (indicated by red lines in Figure 7). Wind also appeared to have interesting patterns with Chl-a. From 8 September, a high wind speed event peaked at 17 m s^−1^ in Trasimeno and remained relatively high, being accompanied by declining and more variable Chl-a concentrations. With the return of calm conditions around 22 September Chl-a immediately appeared to increase. Higher wind speeds with an associated drop in Chl-a were also found in the Curonian Lagoon during this period, although Chl-a concentrations only showed a relatively minor increase with the return of calmer weather around 22 September when temperatures had already dropped to 14.5 °C (Figure 9). Unfortunately, Chl-a was not available for this period for lake Võrtsjärv. In Võrtsjärv and the Curonian Lagoon, there were fewer clear patterns with precipitation which was always below 20 mm per day. All lakes showed an increase in Chl-a in the first two weeks of September when the maxima were recorded for all three lakes for the time period examined.

NPMR was used over this summer period to investigate the influence of weather on the seasonal pattern of Chl-a (Table 3). The day of year was the most sensitive parameter in the analysis for the three lakes, as would be expected given the importance of seasonal variation and antecedent concentrations. Weather parameters that were incorporated into the models included wind speed for Lake Trasimeno, but this was interchangeable with the seven-day antecedent sum of rainfall or solar irradiance with no loss in xR^2^. For Võrtsjärv, the air temperature was significant in the model (interchangeable with daily rainfall or the seven-day antecedent u-vector for wind with a 1% lower xR^2^). The xR^2^ was notably lower for Võrtsjärv at 0.52 compared to the other lakes (≥0.87). For the Curonian Lagoon, the seven-day antecedent air temperature was significant in the model (interchangeable with daily or seven-day antecedent v-vector for wind with a 2% lower xR^2^).

## 5. Discussion

The analysis of daily Chl-a patterns revealed five clusters with two main patterns—either that of a concave shape with a decline reaching a minimum around midday before increasing (clusters 13,42,48), or a convex shape increasing to a peak at 11:00 or 12:00 before declining (cluster 1 and 6). The univariate box-plot analysis indicated that those clusters containing Chl-a patterns that declined towards mid-day typically had higher solar irradiance. This would suggest that the phenomenon of non-photochemical quenching (NPQ) may in operation, whereby an excess of light leads to a decline in the fluorescence quantum yield [73]. This often occurs in shallow waters that typically have higher irradiance [74]. These clusters with the higher solar irradiance contained a mix of lakes with the exception of cluster 42, which was comprised of 90% Lake Trasimeno in Italy, and being 1942 km south of Lake Võrtsjärv, receives higher irradiation (Supplementary Materials Figure S2). Interestingly, as well as having high solar irradiation, this group of clusters typically had lower wind speed (with the exception of cluster 13). This may indicate that this pattern of diurnal decrease in Chl-a towards mid-day may be more active or visible when there is less mixing by wind. The application of multinominal logistic regression was not very successful in the reclassification of the clusters using environmental variables. There may be several reasons for this—perhaps the diurnal pattern of Chl-a is more of a continuum and not amenable to discreet classification, despite the approach being useful in understanding general patterns and their causes. In addition, other unmeasured parameters could have improved the classification. For example, as the patterns appeared to be strongly influenced by solar irradiance then subsurface light profiles would be useful to quantify light attenuation by in water factors such as turbidity and humic substances.

The first event visible in the time series was the temperature increase from the European heatwave in July 2019 followed by the influence of the storm that ended it. The influence of the storm on Chl-a was more evident than the direct influence of the heatwave itself. This may be because the influence of storms is more sudden and therefore visible in time-series. It may also be a question of timing and phenology; the heatwave of 2003, for example, occurred around mid-August and resulted in a *Microcystis* bloom [37]. For Lake Trasimeno, the counts of cyanobacteria from the regional authority showed peak concentrations from 27 August to 2 September 2019 [51]. If the heatwave had occurred later, it may have been accompanied by larger increases in cyanobacteria more frequent during this period and also because they have a temperature optima above 20 °C [36,37]. In addition, if deeper nutrient-rich polymictic lakes had been included in this study there may have been a clearer direct response in Chl-a to the heatwave as stratification, warming, and low wind in these lakes can catalyze blooms [75]. The air temperature decline at the end of the heatwave was up to 19.8 °C. If heatwaves are interrupted by cooling periods with low irradiance, particularly in shallow lakes, the sudden decline in temperature can disrupt metabolism, lower oxygen concentrations, and lead to fish kills [76]. However, high frequency data was not available for oxygen and other parameters for this study to further explore the implications of this event.

After the storm event, the Chl-a increased in both Trasimeno and Võrtsjärv. For Trasimeno, there is a possibility that this was associated with nutrient input from the catchment given the high rainfall. Previously for this lake, satellite images have indicated that riverine inputs stimulated a bloom in 2018 that spread across the lake [28]. In contrast, for Lake Võrtsjärv, while the storm event led to a dramatic drop in temperature that was accompanied by increased wind speed, there was only minor rainfall. The subsequent increase in Chl-a may therefore be associated with nutrient additions from internal loading. A combination of calm warm weather, such as occurred with this heatwave, can increase the release of inorganic phosphorus from sediments as can direct resuspension following wind events [77]. In fact, despite reduced external loading, Lake Võrtsjärv has not shown a decrease in phytoplankton and this has been attributed to phosphorus stored in the sediments from decades of excess fertilizer application in the catchment and wastewater inputs [60]. A similar process of internal loading is also likely to contribute to the Chl-a increase in Lake Trasimeno [47]. In the Curonian Lagoon, the response to the heatwave and subsequent storm was muted and the increase in Chl-a continued through this period from mid-July to mid-August unperturbed. In fact, although the air temperature dropped there was little change in precipitation or wind-speed noted.

A second rainfall event on 21 August appeared restricted to Lake Trasimeno, where it increased Chl-a concentration. Subsequent to this, all of the lakes had a Chl-a maxima in the first two weeks of September, coincident with the typical period of maximum abundance of cyanobacteria, which was confirmed by available microscopic count data for Trasimeno [51]. This was followed a windy period until the 22 September. This had the effect of lowering Chl-a concentrations in Lake Trasimeno and in the Curonian Lagoon (no data was available for Võrtsjärv). Probably, this wind event dissipated the cyanobacteria blooms that had formed in calmer weather, under which they have a competitive advantage [37]. When calmer weather returned around 22 September, the Chl-a increased rapidly in Lake Trasimeno. This could in part be the result of positively buoyant cyanobacteria moving towards the surface and being detected by the WISPstation, although count data of phytoplankton indicate lower cyanobacteria during this time [51], so it may also represent new growth by different taxa or horizontal transport [28]. In the case of the Curonian lagoon, in addition to this process, there may also be a possibility that the increase in wind led to a dilution of the Chl-a in the lagoon with water from the Baltic Sea. When northerly winds increase the level of the Baltic relative to the lagoon, especially at low discharge rates, intrusions can reach the central part of the lagoon [78].

Extracting the response to a heatwave event across several lakes of different nutrient status may be challenging. Mesocosm experiments simulating a heatwave event across very high (>0.300 mg L^−1^ TP) and low nutrient (<0.014 mg L^−1^ TP) treatments found that higher nutrient levels were the key factor determining higher Chl-a, biomass, and gross primary production as well as determining phytoplankton taxonomic composition [76,79]. However, within the low nutrient treatment, biomass and gross ecosystem primary production increased both during and after the heatwave. In contrast, the response of the very high nutrient treatment to the heatwave was less clear. This indicates that heatwaves are likely to have an important influence on ecosystem processes as well as Chl-a, but the response may be harder to isolate at higher nutrient levels.

A recent review of storm impacts on phytoplankton found an inconsistent response of Chl-a which could either increase or decrease, suggesting the importance of specific context in determining the response. Increased understanding requires a detailed characterization of interacting catchments, lakes, and storms as well as antecedent conditions. However, a relatively low sample size was available for the review [80]. The current work should therefore make a contribution to documenting and understanding the response of shallow lakes to heatwaves and storms.

In the NPMR analysis, the day of year was the most important in prediction of Chl-a owing to the importance of seasonal variation and antecedent concentrations, after which the wind speed or temperature was important for the three lakes. However, given that changes in most of the meteorological variables are coincident, it was not surprising that other variables such as rainfall or solar irradiance were interchangeable. It is noteworthy that Lake Võrtsjärv had a lower xR^2^ than the other lakes and the poorer fit may indicate the importance of additional factors unaccounted for. One of these may be that phytoplankton has been reported to be light limited in this lake which can restrict growth [81].

The high frequency monitoring by the WISPstation, organized in a spatial network like this, can be a key resource in observing the influence of climate events at continental scale. Contrasting similar and differing responses among lakes to heatwaves and storms allows the influence of the events to be more clearly visualized. The Chl-a increase after the storm that ended the heatwave was observed in two lakes over 1942 km apart and the event marked a step-change to elevated concentrations that continued for the summer. While concentrations are likely to have increased anyway with the progression of summer, the timing of meteorological events punctuated the shift. This is likely to have significant implications for phenology and lake ecology which needs more detailed research. An additional benefit of using an in situ spectroradiometer like the WISPstation is that it can be used to validate and calibrate satellite data. In this way, remote sensing data can be used to produce Chl-a maps for water quality monitoring going beyond the limited spatial coverage of in situ sampling with synergistic management benefits [26,27,30,31].

High frequency monitoring can improve confidence and precision and help us understand the response of the European water resource to short term rapid climate-driven fluctuations in water quality. It has the key benefit of allowing the temporal scale of monitoring to be set to match that of the ecological process of interest revealing the short and long term impacts of disturbance events providing key inputs to models and timely information for management [82]. While it cannot replace ecological assessment for the WFD that requires taxonomic analysis [82], member states have agreed that climate related threats should be incorporated into river basin management plans and that they should ensure monitoring programs allow for an early detection of the signal of climate impact. This is likely to be more important in future as several member states have already listed extreme climatic events such as floods and droughts as reasons leading to a temporary deterioration of status [83]. The additional benefits of installing high frequency monitoring include early warning for harmful algal blooms, managing recreational use and improving trust for drinking water supplies. However, the relative cost-benefits of these are lake-specific, increasing with the number of users of the lake [84]. An additional example of a practical use of this approach is that catchment and lake managers could mark the period between the end of a heatwave and arrival of a storm as a nutrient sensitive period, whereby nutrient addition to agricultural lands is restricted which can reduce nutrient export under scenarios where rainfall in not very intense [85]. In addition, the construction of ponds to intercept such runoff from agricultural land is also a beneficial solution [86].

Like previous heatwaves observed in Europe, the one that occurred in July 2019 was caused by an ‘omega’ block [45,87], named after the Greek letter Ω, due to the resulting shape formed by the high pressure system in central Europe blocked either side by low pressure systems in the west and east for an extended period of time, producing the heatwave. The increasing frequency of heatwaves in Europe have been linked to the reduction in Arctic sea ice and Eurasian snow cover and are likely to continue during the next century [88]. Thus, it would prudent to increase and interlink high frequency monitoring systems throughout Europe to better understand the environmental implications of climate change. A future examination of this and other heatwave events is planned using the European Space Agency’s Lakes Climate Change Initiative dataset scheduled to expand to 2000 lakes in late 2021 [89]. This dataset contains satellite estimates of lake temperature and chlorophyll-a and could be used to test for responses to heatwave events and storms. Combining such synoptic satellite datasets with high frequency in situ data such as that used in this study may further deepen our understanding. Future research should strive to integrate and understand interrelationships between a catchment, its lakes (including physical, chemical, and biological/phenological characteristics) and heatwaves and antecedent and subsequent weather events [80].

## 6. Conclusions

We found that daily patterns of Chl-a that declined towards mid-day tended to be characterized by high irradiance and low wind.

Heatwaves followed by storms were found to promote blooms in two shallow lakes (Trasimeno and Võrtsjärv), whereas in the Curonian lagoon the response was more muted. The mechanisms for this may be complex and lake specific, either through increased internal and/or external loading delivering nutrients to primary producers already experiencing close to optimum growth temperatures. Such events can punctuate the lakes’ phenological patterns, leading to elevated concentrations of Chl-a for the summer with likely implications for ecosystem functioning as well as lake management. The fact that two of the lakes examined had a similar response to the heatwave and subsequent storm, despite being 1942 km apart, indicates that such events can have a pervasive influence on seasonal dynamics at continental scale. The timing of the heatwave towards the end of July is likely to have been key to the phytoplankton response with lower concentrations of Chl-a than if the heatwave had occurred in late August. The higher winds in September acted to dissipate blooms.

High frequency monitoring using WISPstations was successful in indicating responses at a pan-European level; the first time several in situ spectroradiometers were used for this purpose. As many pressures and climatic drivers operate at global-regional scales, further insight can be gained by networking and using satellite remote sensing to provide a synoptic analysis.

## Figures and Tables

**Table 1:** Main features of Lake Trasimeno, Curonian Lagoon and Lake Võrtsjärv.

Characteristics	Trasimeno	Võrtsjärv	Curonian Lagoon
Catchment area (km^2^)	383	3100	97928
Lake surface area (km^2^)	121	270	1584
Maximum depth (m)	5.5	6	5.8 *
Average depth (m)	4.0	2.8	3.8
Water residence time	>20 years	~1 year	10–100 days
Total phosphorous (µg L^−1^)	27	40	108

* The artificially deepened Klaipeda Strait for harbor activities is 8–15 m in depth.

**Table 2:** Percentage correct classification for multinomial logistic regression of cluster membership using environmental variables (wind speed, solar irradiance, and lake). Percentage correct classification in bold on diagonal. n = number of days in each cluster.

Cluster	1	6	13	42	48	n
1	**56**	47	26	0	0	32
6	3	**0**	2	0	0	17
13	41	53	**66**	80	100	50
42	0	0	6	**20**	0	10
48	0	0	0	0	**0**	11

**Table 3:** Results of NPMR (Nonparametric Multiplicative Regression) models for chlorophyll-a. xR^2^ = cross-validated R^2^; Ave. size = Average neighborhood size; Tol. = Tolerance; Sen. = Sensitivity; Temp_7day = 7-day antecedent average air temperature; Temp = Daily air temperature; DOY = Day of Year.

Lake	xR^2^	Ave. Size	Variable1	Sen.	Tol.	Variable2	Sen.	Tol.	*p*
Curonian	0.91	3.83	Temp_7day	0.29	0.68	DOY	0.56	3.45	<0.05
Võrtsjärv	0.52	5.37	Temp	0.09	2.68	DOY	0.68	3.45	<0.05
Trasimeno	0.87	5.13	Wind speed	0.10	1.63	DOY	0.75	3.45	<0.05

## Data Availability

The datasets generated during and/or analyzed during the current study are available from the corresponding author on reasonable request.

## Supplementary Materials

The following are available online at https://www.mdpi.com/article/10.3390/s21186242/s1, Figure S1: Results of a validation exercise carried out across the three countries for 29 sets of Chl-a estimates derived from the WISPstation and from laboratory measurements (further details in [63]), Figure S2: Results of cluster analysis on daily pattern of chlorophyll-a. Row code refers to date (YYMMDD) and lake: T = Trase-meno, V = Võrtsjärv, C = Curonian Lagoon, coloured by cluster membership.
