# Supplementary material for: Detecting Climate Driven Changes in Chlorophyll-a Using High Frequency Monitoring: The Impact of the 2019 European Heatwave in Three Contrasting Aquatic Systems
Source: Sensors (Basel). 2021 Sep 17;21(18):6242. doi: 10.3390/s21186242 (PMC8473262; doi:10.3390/s21186242)
Supplement: Supplementary file 1 [file sensors-21-06242-s001.zip › sensors-1350559-supplementary.pdf]

sensors

Detecting Climate Driven Changes in Chlorophyll-a Using High Frequency Monitoring: The Impact of the 2019 European Heatwave in Three Contrasting Aquatic Systems

Gary Free [1,*], Mariano Bresciani [1], Monica Pinardi [1], Claudia Giardino [1], Krista Alikas [2], Kersti Kangro [2,3] Eva-Ingrid Rõõm [3], Diana Vaičiūtė [4], Martynas Bučas [4], Edvinas Tiškus [4], Annelies Hommersom [5], Marnix Laanen [5] and Steef Peters [5]

Supplementary material

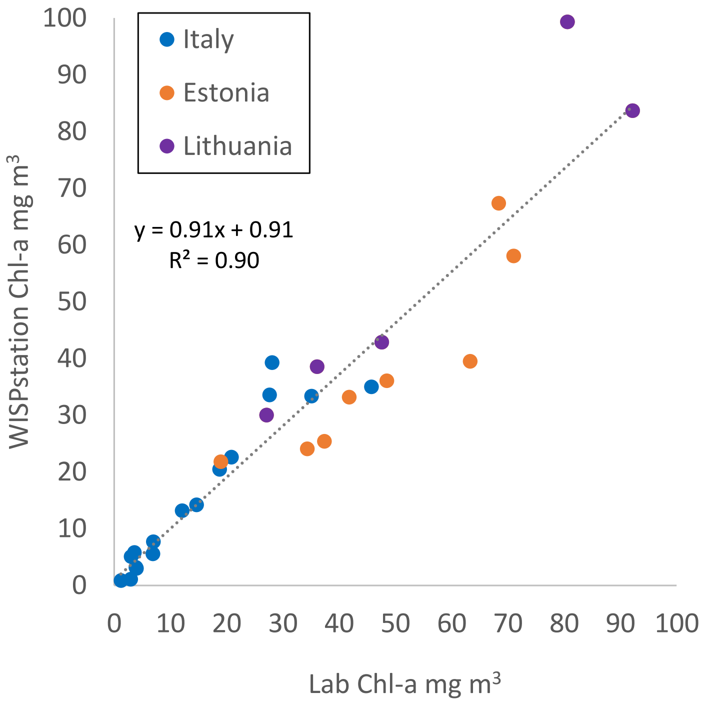

Figure S1. Results of a validation exercise carried out across the three countries for 29 sets of Chl-a estimates derived from the WISPstation and from laboratory measurements (further details in [63]).

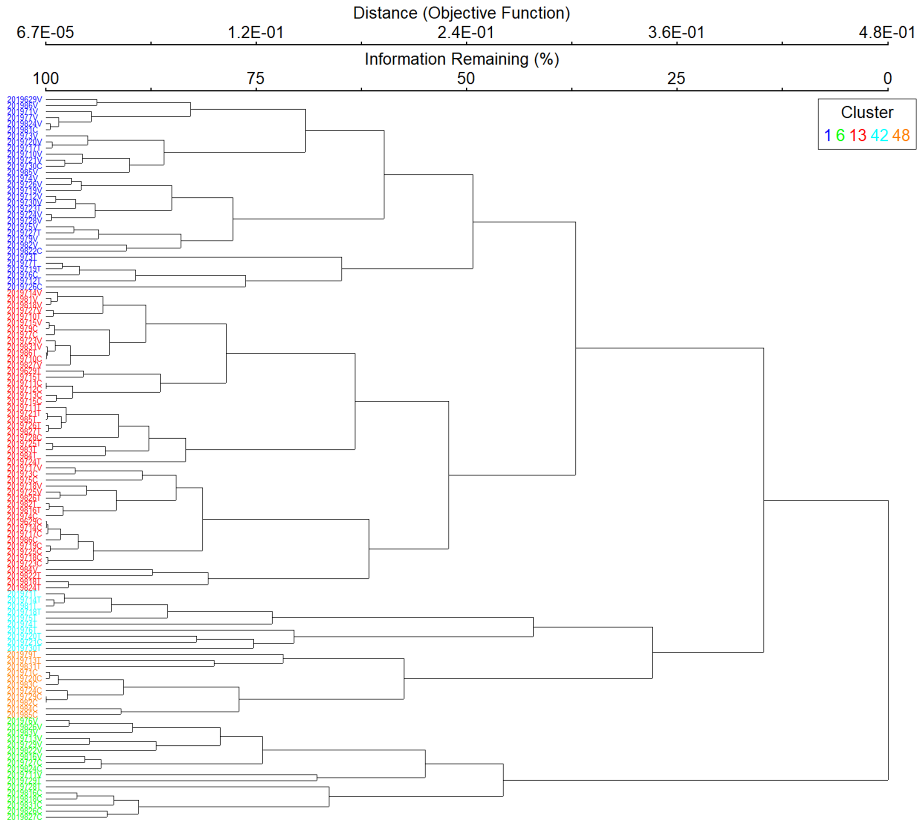

Figure S2. Results of cluster analysis on daily pattern of chlorophyll-a. Row code refers to date (YYMMDD) and lake: T = Trasemeno, V = Võrtsjärv, C = Curonian Lagoon, coloured by cluster membership.
